# The Importance of Radiologic Imaging Modalities in Autosomal Dominant Polycystic Kidney Disease

**DOI:** 10.7759/cureus.31480

**Published:** 2022-11-14

**Authors:** Jorge Nadal Bosch, Javier Malcolm, Mario Moya, Michael Menowsky, Paul Dominici

**Affiliations:** 1 Internal Medicine, Doctors Hospital at Renaissance/University of Texas Rio Grande Valley, Edinburg, USA; 2 Medical Information, Doctors Hospital at Renaissance, Edinburg, USA; 3 Radiology, Doctors Hospital at Renaissance/University of Texas Rio Grande Valley, Edinburg, USA; 4 Emergency Medicine/Critical Care, Doctors Hospital at Renaissance/University of Texas Rio Grande Valley, Edinburg, USA; 5 Emergency Medicine, Doctors Hospital at Renaissance/University of Texas Rio Grande Valley, Edinburg, USA

**Keywords:** extra-renal manifestation of adpkd, imaging modalities, gallium scan, cyst infection, adpkd

## Abstract

Autosomal dominant polycystic kidney disease (ADPKD) is a common disorder that occurs in approximately one in 1000 live births. Patients may be asymptomatic or present with symptoms such as hypertension, hematuria, proteinuria, or renal function impairment. It can present with extra renal complications like cerebral aneurysms, hepatic and pancreatic cysts, infected cysts, cardiac valve disease, colonic diverticula, abdominal wall and inguinal hernia, and seminal vesicle cyst. Imaging studies such as ultrasonography (US), computed tomography (CT), and magnetic resonance imaging (MRI) provide vital information regarding the diagnosis of the disease, monitoring of the progression of the disease, and detection of complications from the disease. We present the case of a 40-year-old male who developed extra-renal complications, and how different imaging modalities facilitated and enabled us to optimize the care of this patient in a timely manner.

## Introduction

Autosomal dominant polycystic kidney disease (ADPKD) is a systemic disorder characterized by the progressive expansion of multiple bilateral renal cysts leading to enlargement and distortion of the kidney which causes chronic kidney disease, hematuria, pain, infected cysts, and extra-renal complications. ADPKD is the most common genetic disorder which occurs in approximately one in 1000 live births and is one of the most common causes of end-stage renal disease (7-8%) [1]. ADPKD is a heterogenetic disorder caused by mutations in PKD1 which is located in chromosome region 16p13.3 and PKD2 located in chromosome region 4q21 [2]. Renal cysts are the most common clinical manifestation, but extra renal cysts may arise in the liver, pancreas, spleen, ovaries, and arachnoid matter.

## Case presentation

A 40-year-old gentleman with a past medical history of hypertension presented with shortness of breath, abdominal pain, decreased urine output, and edema. The patient reported that shortness of breath started four days ago and has been progressively worsening. Abdominal pain was localized in both flanks, characterized as dull, and constant, the intensity was 9/10, alleviated by acetaminophen, no aggravating factors, associated with decreased urine output and edema. The patient's vital signs were a temperature of 97.5°F, heart rate of 116, respiratory rate of 22, blood pressure of 114/67 mmHg, and oxygen saturation of 98% on room air. On physical examination, the patient was found alert and oriented to person, time, and place. Lungs were clear to auscultation, respirations were nonlabored. The cardiovascular exam was a normal rate, normal rhythm, no murmur, and trace edema in the lower extremity bilaterally. The gastrointestinal exam was soft, with diffuse tenderness all over four quadrants, mildly distended, and normal bowel sounds. The genitourinary exam was positive for costovertebral angle tenderness. The neurological exam was normal with no focal defects, and cranial nerves 2-12 were grossly intact.

**Figure 1:**
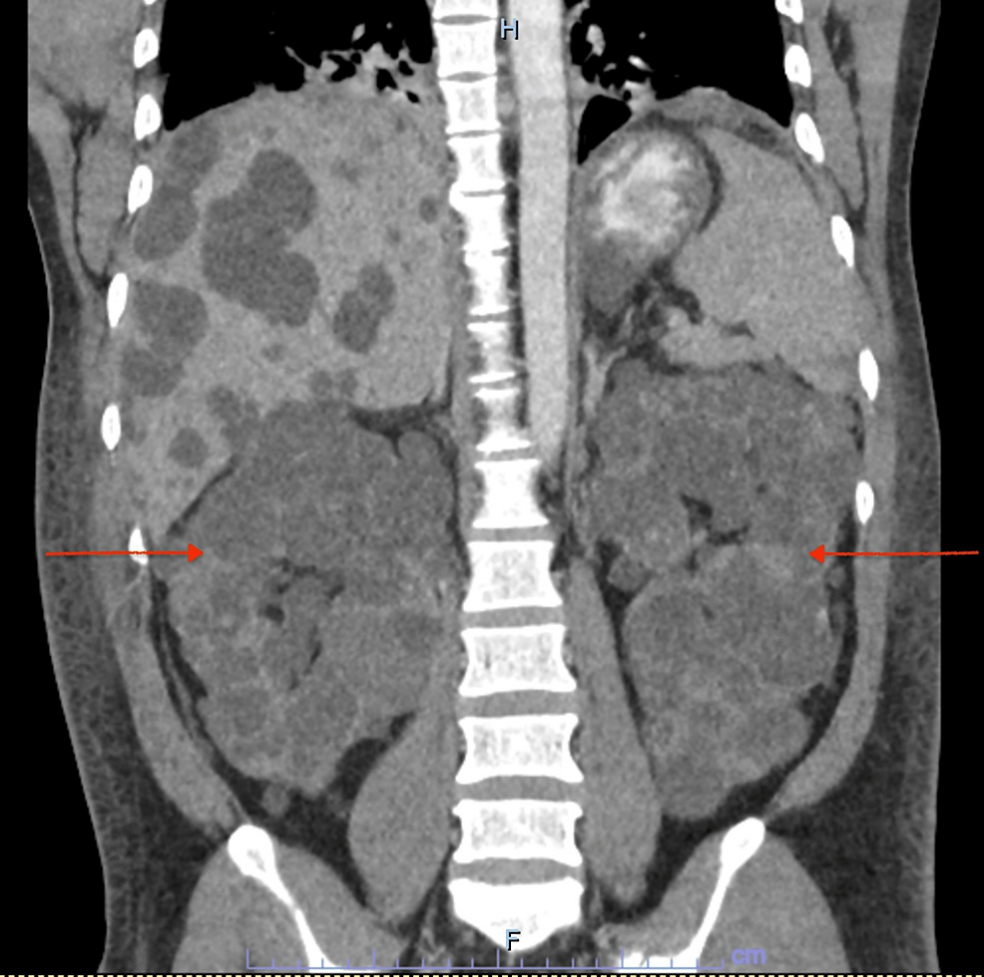
Computed tomography of the abdomen and pelvis without contrast showing bilateral renal cysts (arrows)

**Figure 2:**
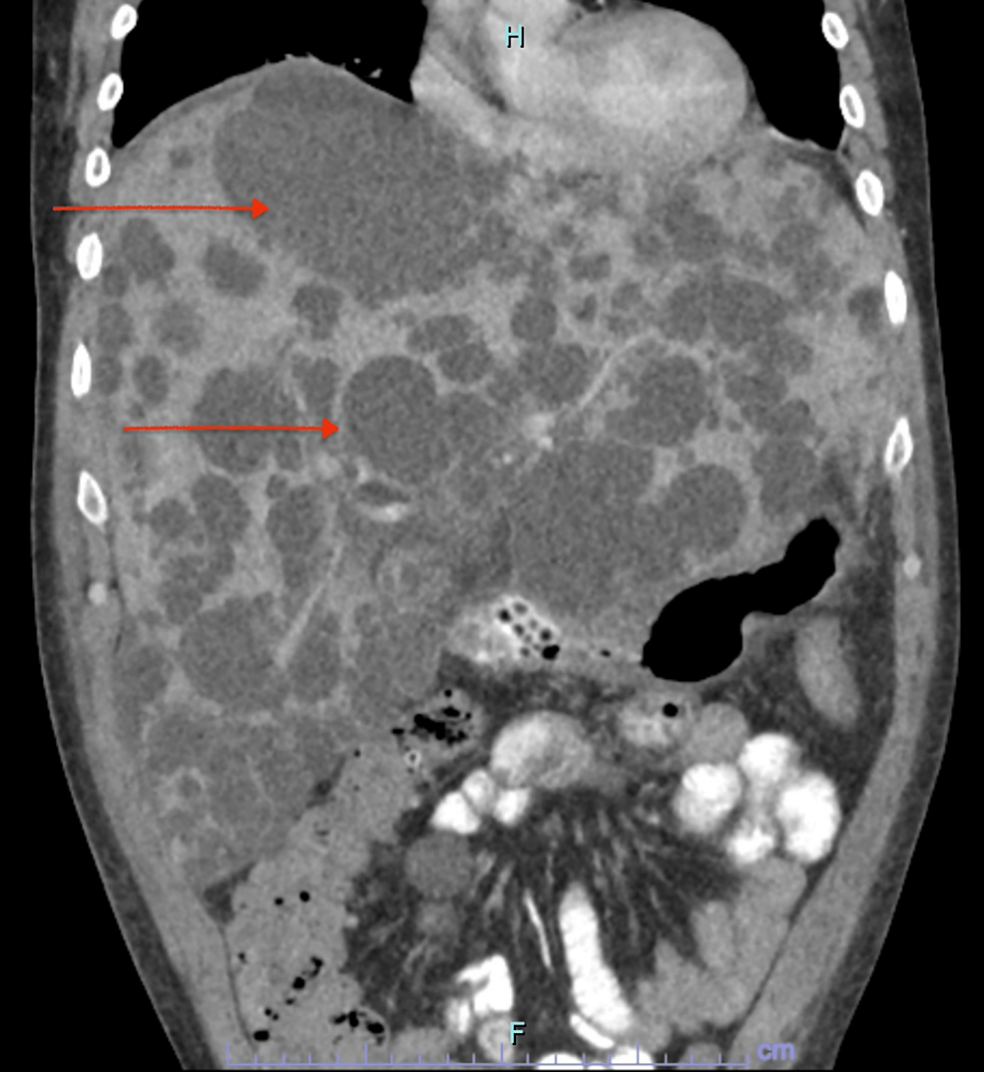
Computed tomography of the abdomen and pelvis without contrast showing liver cysts (arrows)

Laboratory studies revealed WBC 13.7 x 10^3^/uL, hemoglobin 11.0 g/dL, hematocrit 31.8%, mean corpuscular volume (MCV) 81.1 fL, platelets 65 x 10^3^/uL, sodium 133 mmol/L, potassium 3.4 mmol/L, chloride 100 mmol/L, carbon dioxide 7 mmol/L, creatinine 19.1 mg/dL, blood urea nitrogen (BUN) 195 mg/dL, estimated glomerular filtration (eGFR) 3 mL/min/1.73 m^2^, calcium 7.9 mg/dL, glucose 144 mg/dL, aspartate aminotransferase (AST) 123 IU/L, alanine transaminase (ALT) 164 IU/L, alkaline phosphatase 124 IU/L, albumin 3.4 g/dL, total bilirubin 1.1 mg/dL, prothrombin time (PT) 16.2 seconds, international normalized ratio (INR) 1.37, partial thromboplastin time (PTT) 31.9 seconds, lactic acid 1.45, alcohol serum level < 10.0 mg/dL, troponin 0.02 ng/mL, and B-type natriuretic peptide (BNP) 66 pg/mL. Arterial blood gas showed arterial pH 7.35, pCO2 14, PO2 111, and HCO3 7.7. Hepatitis A IgM antibody negative, hepatitis-B core total antibody negative, hepatitis-B antigen negative, hepatitis-B surface antibody negative, hepatitis-C virus antibody negative. CT scan of the abdomen and pelvis without contrast showed innumerable bilateral simple cysts measuring up to 5 cm in both kidneys (Figure 1) and innumerable low-density cystic lesions measuring up to 6 cm in the liver (Figure 2).

MRI of the brain without contrast showed no aneurysm. Judging from his symptoms, and the CT scan findings, the most likely diagnosis was ADPKD.

**Figure 3:**
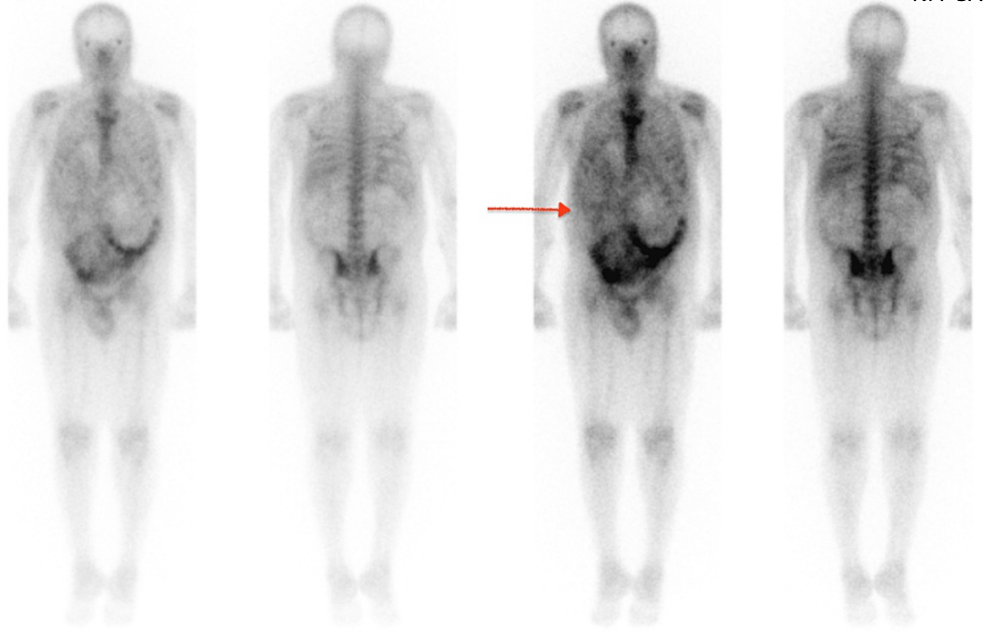
Normal biodistribution of gallium 68 in the kidneys and liver with no signs of focal uptake (arrow)

The patient was started on broad-spectrum antibiotics with aztreonam 1 g IV q24h and meropenem 500 mg IV q12h. Nephrology was consulted for acute renal failure secondary to ADPKD. The patient had placement of a right internal jugular vein Trialysis catheter and was started on hemodialysis. On the third day, two sets of blood cultures grew Escherichia coli and the urine culture grew 10,000-50,000 cfu/mL staphylococcus epidermidis. Aztreonam and meropenem were discontinued and ceftriaxone 2 g IV q24h and metronidazole 500 mg PO were started. The Gallium scan showed a heterogeneous distribution of the tracer in the liver consistent with the patient's known liver cyst (Figure 3), but no evidence of infection or abscess so no clear source of the bacteremia was identified.

Follow-up blood cultures showed no growth. The patient was discharged on oral levofloxacin for three more weeks completing a course of four weeks in total of antibiotics.

## Discussion

In the United States, ADPKD causes renal failure requiring dialysis in 5% of patients [3-4]. ADPKD is caused by mutations in one of two genes known as PKD1 on chromosome 16 which encodes polycystin-1 and PKD2 on chromosome 4 which encodes polycystin-2 [3]. As the disease progresses glomerular filtration rate declines and the average reduction is 4.4 to 5.9 mL/min per year [5]. Risk factors that have been associated with progressive kidney disease in ADPKD are genetic factors, kidney size, hypertension, early onset of symptoms, male gender, proteinuria, and high urinary sodium excretion [6]. Patients who inherit PKD1 or PKD2 mutations will develop kidney cysts that are visible in imaging studies [7]. Clinical manifestations such as kidney function impairment or hypertension are variable based on age. Patients can present with hypertension, hematuria, proteinuria, kidney function impairment, flank pain, obstructive calculi, or urinary tract infection [8]. The most common cause of death in patients with ADPKD is related to heart disease. ADPKD is suspected when patients present with the clinical features mentioned above and in those with a family history. ADPKD diagnosis is confirmed primarily by imaging. The choice of imaging study for the initial evaluation depends on clinical presentation and family history. In a patient with a family history of ADPKD, asymptomatic and normal kidney function an ultrasound is sufficient [9]. If ultrasonography confirms the diagnosis, a baseline CT or MRI is ordered to monitor the disease progression [10]. In a patient with classic findings of ADPKD such as decreased estimated glomerular filtration rate, palpable kidneys, and or family history of ADPKD the preferred imaging to obtain is CT or MRI rather than ultrasound [11]. This will enable the physician to confirm the diagnosis and will serve as a baseline image for comparison and will also help to identify extra-renal disease. The choice between CT or MRI depends on the kidney function of the patient because CT will expose the patient to contrast and will worsen renal function even more and potentially causing contrast-induced acute kidney injury [12]. The initial management for all patients with ADPKD consists of management of blood pressure, dietary sodium restriction, and increased fluid intake unless eGFR is less than 30 mL/min/1.73m^2^. Patients with a high risk for the progression of CKD should be identified because they will benefit from treatment with tolvaptan. The Mayo classification system is the preferred method to identify high-risk patients. It categorizes patients into five classes from low to high risk for disease progression. The Mayo classification system uses the patient’s age, height, and total kidney volume [13]. The total kidney volume can be calculated using a coronal image obtained by CT scan without contrast or MRI without gadolinium. Renal cyst infection is a serious complication of ADPKD and requires early identification in order to manage patients in a timely manner. Gallium scan identifies cells that are dividing quickly detecting inflammation, infection, or cancer. This is one method that can be used in order to confirm an infected cyst [14]. Here we presented a case of a patient who presented with clinical manifestations of ADPKD and had a CT scan that confirmed the diagnosis. He was also found to have bacteremia and there was suspicion of a possible renal or liver-infected cyst, but it was ruled out with the gallium scan. MRI of the brain with contrast ruled out intracranial aneurysm which is also one of the complications of ADPKD.

## Conclusions

We report a case of a patient with the typical clinical presentation of ADPKD with extra-renal manifestation which is a common disorder. This case stresses the importance of knowing that specific imaging modalities support different aspects of care in ADPKD. Radiologic imaging studies provide guidance in the diagnosis and management of these patients. Radiologist interpretation of imaging is essential in the care of ADPKD and facilitates physicians to optimize the care of these patients.
